# Characterisation of *Staphylococcus aureus* isolated from rabbits in Fujian, China

**DOI:** 10.1017/S0950268819001468

**Published:** 2019-08-23

**Authors:** J. Wang, L. Sang, S. Sun, Y. Chen, D. Chen, X. Xie

**Affiliations:** Institute of Animal Husbandry and Veterinary Medicine, Fujian Academy of Agricultural Sciences, Fuzhou, Fujian, People's Republic of China

**Keywords:** Antimicrobial susceptibility, multi-locus sequencing typing, rabbit, virulence factors, *Staphylococcus aureus*

## Abstract

*Staphylococcus aureus* has been recognised as one of the important zoonotic pathogens. However, knowledge about the epidemiology and genetic characteristics of *S. aureus* in rabbits was limited. The aim of this study was to determine the characteristics of 281 *S. aureus* isolated from dead rabbits of nine rabbit farms in Fujian Province, China. All the isolates were characterised by multi-locus sequencing typing, detection of virulence factors and antimicrobial susceptibility test. The results showed that the 281 isolates were grouped into two sequence types, ST121 (13.52%, 38/281) and ST398 (86.48%, 243/281). Surprisingly, the ST121 strains were only recovered from the lung samples from one of the nine rabbit farms studied. In the 281 isolates, the virulence genes of *nuc*, *hla*, *hlb*, *clfA*, *clfB* and *fnbpA* were positive, whereas the *sea*, *seb*, *tsst*, *eta* and *etb* genes were negative. Notably, the 38 ST121 isolates carried the *pvl* gene. All the 281 isolates were methicillin-susceptible *S. aureus*, and the isolates were susceptible to most of the used antibiotics, except for streptomycin, kanamycin, azithromycin and penicillin, and the resistance rates of which were 23.84%, 19.57%, 16.01% and 11.03%, respectively. This study first described the epidemiology and characteristics of *S. aureus* in rabbits in Fujian Province, which will help in tracking the evolution of epidemic strains and preventing the rabbit–human transmission events.

## Introduction

*Staphylococcus aureus* is an important zoonotic pathogen with worldwide distribution. Due to the broad spectrum of virulence factors and the ability to develop antibiotic resistance, the infection of *S. aureus* results in a high morbidity and mortality [1–4]. In humans, *S. aureus* is usually associated with skin and soft-tissue infections, endovascular infections, pneumonia, septic arthritis, endocarditis, osteomyelitis and sepsis [1]. The colonisation of *S. aureus* in animals has been receiving comprehensive attention since animals may potentially act as a reservoir of human infection [5, 6].

Rabbit is one of the most important hosts of *S. aureus*. The infected rabbits are mainly characterised by subcutaneous abscesses, mastitis and pododermatitis [7]. To our knowledge, *S. aureus* is widespread in rabbits in Fujian Province of China. However, knowledge about the epidemiology and characteristics of *S. aureus* in rabbit in Fujian Province is limited. In this study, multi-locus sequencing typing (MLST), tested virulence factors and antimicrobial susceptibility was done on different isolates of *S. aureus* recovered from lesions (pneumonia, mastitis and pododermatitis) in dead rabbits.

## Methods

### Sample collection and *S. aureus* isolation

From August 2017 to December 2018, 466 lung samples, 93 mastitis samples and 132 pododermatitis samples were collected from dead rabbits of nine rabbit farms in three cities (Fuzhou, Longyan and Nanping) of Fujian Province. Each sample was mixed with sterile phosphate buffer saline and homogenised to make 50% suspension. A 100 µl of suspension was inoculated on sheep blood agar plate for cultivation at 37 °C for 24 h. The suspected *S. aureus* colony was initially identified by colony morphology and Gram-staining. The presumptive colony was then sub-cultured and further confirmed by sequencing of *16S rRNA* and *nuc* [8, 9].

### Multi-locus sequencing typing

Genotyping of *S. aureus* isolate was performed by MLST [10]. Briefly, seven housekeeping genes (*arcC*, *aroE*, *glpF*, *gmk*, *pta*, *tpi* and *yqiL*) of *S. aureus* isolate were amplified and sequenced, and sequence type (ST) of the isolate was defined according to the allelic numbers of the seven housekeeping genes (http://www.mlst.net).

### Virulence gene detection

The presence of virulence genes in *S. aureus* isolate was screened by PCR assays using primers previously reported, including thermonuclease (*nuc*) [9], panton-valentine leucocidin (PVL) toxin (*pvl*) [11], entorotoxin (*sea* and *seb*) [12], toxic shock syndrome toxin-1 (*tsst*) [13], exfoliative (*eta* and *etb*) [13], haemolysin (*hla* and *hlb*) [11], clumping factor (*clfA* and *clfB*) [14] and fibronectin-binding protein (*fnbpA* and *fnbpB*) [14]. The PCR products were separated and sequenced.

### Antimicrobial susceptibility testing

The antimicrobial susceptibility of *S. aureus* isolates for 12 antibiotics was performed using the disk diffusion method according to Clinical and Laboratory Standards Institute (CLSI) guidelines [15]. The following antibiotics were used: penicillin, streptomycin, gentamycin, enrofloxacin, kanamycin, ciprofloxacin, cefminox, azithromycin, florfenicol, levofloxacin, ceftizoxime and ceftriaxone. The *S. aureus* ATCC 29213 was used as control. Moreover, the presence of *mecA* or *mecC* gene in *S. aureus* isolate was screened to confirm methicillin-resistant *S. aureus* (MRSA) [16, 17].

## Results

### *S. aureus* isolation and identification

A total of 281 *S. aureus* isolates were recovered from the 691 samples of dead rabbits. Among them, 93 strains were isolated from 466 lung samples, 78 strains from 93 mastitis samples, 110 strains from 132 pododermatitis samples (Table 1).

**Table 1.** Sample collection, *S. aureus* isolation and sequence types of the isolates

Cities	Rabbit farms	No. of samples			No. of isolates			STs
		Lung	Mastitis	Pododermatitis	Lung	Mastitis	Pododermatitis	
Fuzhou	Farm A	105	7	26	38	0	0	121
					8	6	21	398
	Farm B	95	28	14	11	24	13	398
Longyan	Farm C	39	2	8	3	1	5	398
	Farm D	52	8	12	12	7	8	398
	Farm E	50	13	13	4	11	10	398
	Farm F	22	6	15	3	5	13	398
	Farm G	30	10	14	7	9	12	398
Nanping	Farm H	48	11	23	6	7	22	398
	Farm I	25	8	7	1	8	6	398
Total		466	93	132	93	78	110	

### Multi-locus sequencing typing

The genotype of *S. aureus* isolate was determined by MLST. It was shown that the 281 *S. aureus* isolates were divided into two STs, ST121 and ST398. In the genotype of ST121, all of the 38 isolates were only isolated from lung samples collected from Farm A (Table 1). In the genotype of ST398, 55 isolates were recovered from lung samples, 78 isolates from mastitis samples and 110 isolates from pododermatitis samples (Table 1).

**Fig. 1.**
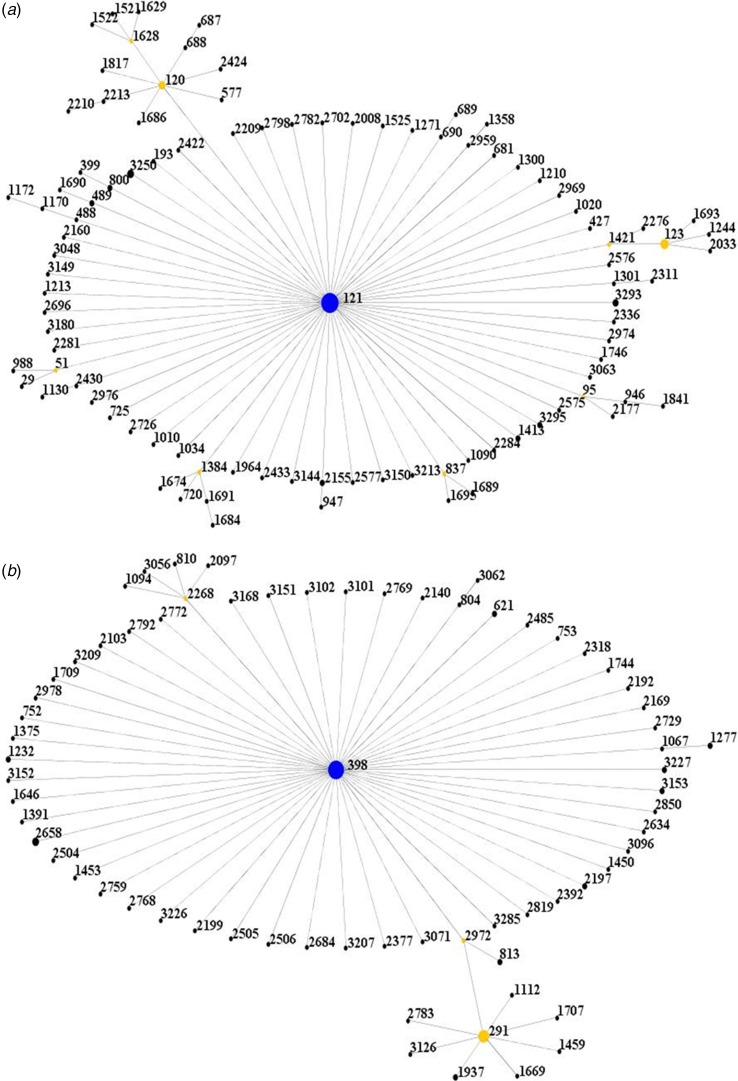
Population snapshot of the isolates. (A) The ST121 is the putative founder of the CC121 and coloured blue. (1B) The ST398 is the putative founder of the CC398 and coloured blue.

All isolates were further clustered into clonal complexes (CCs) by using eBURST software (http://saureus.mlst.net/eBURST/). It was shown that the isolates were clustered into 2 CCs, CC121 and CC398 (Fig. 1).

### Virulence gene detection

All the isolates were positive for the genes *nuc*, *hla*, *hlb*, *clfA*, *clfB* and *fnbpA*, whereas *sea*, *seb*, *tsst*, *eta* and *etb* genes were negatives. The *pvl* gene was detected in the 38 isolates of ST121 isolated from lung samples, and the *fnbpB* gene was positive in 109 isolates of ST398 recovered from lung, mastitis and pododermatitis samples.

### Antimicrobial susceptibility testing

The antimicrobial susceptibility testing showed that all the isolates were susceptible to florfenicol, ceftizoxime and ceftriaxone. The resistance rates to penicillin, streptomycin, kanamycin and azithromycin were more than 10%. Sixteen strains were resistant to ⩾3 antibiotics and the mostly resistant antibiotics were streptomycin, kanamycin and azithromycin (Table 2). Notably, the ST121 isolates showed a lower number of resistances compared with ST398 isolates. Moreover, all isolates were *mecA* and *mecC* negative, indicating that these isolates were methicillin-susceptible *S. aureus* (MSSA).

**Table 2.** Antimicrobial susceptibility profiles of the isolates according to STs

STs	Isolates (*n*)	P (%)	S (%)	GM (%)	ENR (%)	K (%)	CIP (%)	CFM (%)	AZM (%)	FFC (%)	LEV (%)	ZOX (%)	CRO (%)
121	38	0	13.16	2.63	0	5.26	0	0	0	0	0	0	0
398	243	12.76	25.51	11.11	2.88	21.81	4.53	2.06	18.52	0	0	0	0
Total	281	11.03	23.84	9.96	2.49	19.57	3.91	1.78	16.01	0	0	0	0

P, penicillin; S, streptomycin; GM, gentamycin; ENR, enrofloxacin; K, kanamycin; CIP, ciprofloxacin; CFM, cefminox; AZM, azithromycin; FFC, florfenicol; LEV, levofloxacin; ZOX, ceftizoxime; CRO, ceftriaxone.

## Discussion

This is the first study of the characteristics of *S. aureus* from rabbits in Fujian Province in southeastern China. The results showed that *S. aureus* was prevalent in the nine rabbit farms of Fuzhou, Longyan and Nanping. The mortality rates of the nine rabbit farms caused by the infection of *S. aureus* ranged from 18.37% to 52.9%, suggesting that *S. aureus* was the important pathogen causing the death of rabbits in these rabbit farms.

Generally, *S. aureus* has a combination of virulence factors, which were thought to contribute to the pathogenicity [18]. All isolates in this study carried a panel of virulence genes *nuc*, *hla*, *hlb*, *clfA*, *clfB* and *fnbpA*. Moreover, the *pvl* gene was detected in the 38 ST121 isolates, and the *fnbpB* gene was detected in the 109 ST398 isolates. The 38 ST121 isolates that carried the *pvl* gene were all recovered from the lungs of dead rabbits with severe respiratory disease. The severe respiratory disease caused by the infection of the ST121 isolates was an isolated case on a rabbit farm in Fuzhou in late August 2017. The infection of the isolate caused the death of about 1000 rabbits in a 4-week period [14]. It was revealed that the PVL was related to necrotising pneumonia [19, 20]. Infection of both PVL-positive MRSA and MSSA could lead to necrotising pneumonia [21], indicating that the PVL of the 38 ST121 isolates might be one of the crucial factors contributing to the severe respiratory diseases. The fibronectin-binding proteins (FnBPA and FnBPB) are multifunctional virulence factors, which facilitate *S. aureus* colonisation and invasion of the host cells [18, 22]. In consistent with the previous reports, all ST398 isolates in this study expressed at least one fibronectin-binding protein, while some of the isolates expressed both FnBPA and FnBPB. A previous study showed that five out of the six ST398 strains isolated from raw milk of dairy cows with mastitis were positive of both *fnbpA* and *fnbpB*, while the other one isolate only carried *fnbpA* [23]. However, an MRSA ST398 strain from urinary tract infection in a child only carried *fnbpB* [24]. Further studies are needed to understand the pathogenic mechanisms of FnBPB in the FnBPB-positive strains isolated from rabbits.

The 281 isolates in this study were only grouped into two STs, ST121 and ST398. Surprisingly, the ST121 strains, but not the ST398 strains, were also detected in a tertiary referral hospital in Xiamen city of Fujian Province [25]. Compared to previous reports, strains belonged to other STs were also isolated in rabbits from Spanish, Thailand and Iberian Peninsula [26–28], indicating that the STs of *S. aureus* in rabbits might vary geographically. *S. aureu*s strains that belong to ST121 and ST398 were worldwide distributed, and have broad host spectra including both humans and animals. Commonly, ST121 isolates are MSSA that are susceptible to methicillin [28, 29]. In contrast to ST121, strains of ST398 could be further grouped into healthcare-associated, community-associated and livestock-associated strains [1, 2, 5, 6]. Among them, the livestock-associated MRSA ST398 was highly concerned for the transmission to humans from animals [5]. Transmission of livestock-associated MRSA from swine and bovine to humans had been documented previously [6, 30]. The case of livestock-associated MRSA ST398 occurring in rabbits and involving farm workers or their family members was also reported in a rabbit farm in Italy [31]. According to previous reports, *S. aureus* frequently acquired antimicrobial resistance [2–4]. Although isolates resistant to ⩾3 antibiotics were detected in this study, the isolates were all MSSA and no MRSA was detected.

Fujian Province was recognised as one of the key areas for rabbit breeding in China. With the rapid development of rabbit farming in Fujian Province, more studies are needed to understand the epidemiology and characteristics of *S. aureus* in rabbits and to elucidate its relationship with the human.
